# 

**Published:** 1904-05-14

**Authors:** 


### NOTICE TO CORRESPONDENTS.

All MSS., Letters, Books for Review, and other matters intended for the Editor should be addressed The Editor, "The Hospital," The Hospital Buildings, 28 & 29 Southampton Street, Strand, W.C.

The Editor cannot undertake to return rejected MS., even when accompanied by stamped directed envelope.

**Notice to Advertisers and Subscribers.**

All Advertisements, Orders for Copies of The Hospital, and Business Communications should be addressed to **THE MANAGER (Not to the Editor),** The Hospital, 28 & 29 Southampton Street, Strand, London, W.C.

The Cost of Subscription, post free, is as follows:—Per week, 3½d.; per quarter, 3s. 3d.; per half-year, 6s. 6d.; per annum, 13s. Foreign and Colonial:—Per annum, 19s. 6d., payable, in all cases, in advance.

**NOTICE.—Vol. XXXV. of "The Hospital" (October 1903 to March 1904), in handsome cloth binding, is now on sale at the office of this Journal, price 10s. 6d. Cases for binding the Numbers contained in this Volume 2s. Index to Vol. XXXV., 3½d., post free.**

**The Monthly part for May is now ready. Price 1s., by post, 1s. 3d.**

**PADDINGTON GREEN CHILDREN'S HOSPITAL, London, W.**

(Convalescent Home, Fair View, Slough, Bucks.)

Notice is hereby Given that the TWENTY-FIRST ANNUAL MEETING of Contributors to the funds of this Hospital will be held at the Hospital, Paddington Green, on Thursday, 19th May, at Five o'clock p.m., for the following purposes:—To receive the Report and Statement of Accounts for the year ended 31st December, 1903; to elect members of Committee in place of those retiring; and to appoint Auditors for the current year.

The Chair will be taken by Douglas Owen, Esq., who will be supported by the Hon. W. H. Goschen and George Hanbury, Esq.

By Order,

W. H. PEARCE,

Secretary.

11th May, 1904.

N.B.—The attendance of ladies is especially invited.

### THE STUDENT OF MEDICINE.

### APPOINTMENTS.

J. W. Adam, M.B., C.M.Glasg., Medical Officer of Health to the Aberdeen Town Council. **Wilfred A. Aldred**, M.R.C.S., L.R.C.P., House Surgeon at the Kent and Canterbury Hospital. **William Clarke**, M.B.Toronto, Clinical Assistant to the Chelsea Hospital for Women. **F. S. Hardy**, M.R.C.S, L.R.C.P.Lond., District Medical Officer of the Sheffield Union. **Charles A. Hayman**, M.D.St.And., F.R.C.S.I., L.D.S.R.C.S., Assistant Dental Surgeon to the Bristol Royal Infirmary. **H. A. Hinds**, M.R.C.S., L.R.C.P.Lond., District Medical Officer of the Bridge Union. **H. W. Jackson**, M.D.Lond., L.R.C.P., M.R.C.S., D.P.H., Surgeon to the North Ormesby Hospital, Middlesbrough. **F. C. James**, M.B.Durh., D.P.H., Medical Officer of Health Hoxne Rural District and District Medical Officer and Public Vaccinator for the Fressingfield District. **A. Thomson**, M.B., District Medical Officer of the Barnsley Union. **P. D. Turner**, M.D., Medical Officer of Health for the Borough of Ryde.

**"The Hospital" Institutional Library.**

"Hospitals and Asylums of the World." 4 Vols., with a Portfolio of Plans, complete £12 12s.

"Burdett's Hospitals and Charities, 1903." 5s. net.

"Burdett's Official Nursing Directory, 1903." 3s. net.

"Cottage Hospitals, General, Fever, and Convalescent." 10s. 6d.

"Uniform System of Accounts for Hospitals and Public Institutions." New Edition. 4s. net.

"Hospital Expenditure: The Commissariat." 2s. 6d.

"A Legal Handbook for the Use of Hospital Authorities." 2s. 6d.

"The Cottage Hospital Case Book or Register of Patients." 10s. and 12s. 6d.

All these are published by the Scientific Press, Ltd., and may be obtained through any bookseller, or direct from the publishers 28 and 29 Southampton Street, Strand, London, W.C.

### Important Notice to our Readers.

Owing to the many representations we have received from Nurses, whose duties take them into remote country districts, as to the difficulties they experience in procuring The Hospital locally, it has been decided, in order that Nurses situate far removed from Towns may not be thus inconvenienced, to supply The Hospital direct, by first post on Friday morning as far as possible, without any charge for postage.

**SUBSCRIPTION RATES for United Kingdom only:—**

| | |
|---|---|
| **One Year... ... ... ...** | **13s. 0d. post free.** |
| **Six Months ... ... ...** | **6s. 6d. „** |
| **Three Months ... ... ...** | **3s. 3d. „** |

For a period of less than three months $3\frac{1}{2}d.$, including postage, will still be charged. Therefore it is necessary, in order to secure the advantage of these special facilities, to subscribe for at least three months.

N.B.—This new arrangement does not apply to subscriptions at present existing, but present subscribers will have the advantages of these rates when renewing their subscriptions.

Address THE MANAGER, "THE HOSPITAL,"
The Hospital Building, 28 & 29 Southampton Street,
Strand, London, W.C

